# Streptococcus intermedius: From a Normal Oral Commensal to a Life-Threatening Organism

**DOI:** 10.7759/cureus.50708

**Published:** 2023-12-18

**Authors:** Anas Mahmoud, Tala Beilani, Nizar Alyassin, Karam Zakharia, Taha Basil, David Poulad

**Affiliations:** 1 Internal Medicine, St. Joseph’s University Medical Center, Paterson, USA; 2 Oncology, Kansas City University, Kansas City, USA; 3 Infectious Disease, St. Joseph’s University Medical Center, Paterson, USA; 4 Neurosurgery, St. Joseph’s University Medical Center, Paterson, USA

**Keywords:** brain abscess, craniotomy, diabetes mellitus, emergency medicine, medical intensive care unit (micu), neurosurgery, seizure, skin abscess, streptococcus intermedius bacteremia, subdural empyema

## Abstract

Subdural empyema is a collection of pus in the subdural space between the dura mater and the arachnoid. It carries very high morbidity and mortality as it can spread anywhere in the brain; however, the risk can be mitigated with appropriate surgical and medical intervention. Being protected by the skull, cranial infections are usually preceded by a significant risk factor, either an external invader such as skull fractures secondary to trauma, penetrating injury, prior surgery, or, more commonly, in more than 50% of cases, due to spread of an internal infection such as ear or sinus infections. Anaerobic and aerobic bacteria can cause subdural empyema. Both gram-positive and gram-negative bacteria are notorious for developing this kind of infection; for example, different groups of gram-positive streptococci and staphylococci, gram-negative *Haemophilus influenza*, and other gram-negative bacilli can cause subdural empyema. While streptococci are more frequent with sinus infection causing subdural empyema, staphylococci are associated with skin invasion secondary to either head trauma or cranial surgery. *Streptococcus intermedius* is a gram-positive alpha-hemolytic pathogen belonging to the larger *Streptococcus anginosus* group that itself is a subgroup from viridans streptococci, aka *Streptococcus milleri*. S*treptococcus intermedius* is an oral commensal flora and is considered to be a low-virulence bacteria in immunocompetent patients but can be associated with significant morbidity and mortality. Subdural empyema tends to occur more often in immunocompromised patients such as diabetic patients, those with human immunodeficiency virus infection, and those using immunosuppressive medications. The clinical course ranges from indolent to fulminant. The size and location of the abscess play a role in clinical presentation. Headache is the most common presenting symptom, but patients can also present with fever, nausea, seizure, or altered mental status. Diagnosis can be obtained with CT and MRI scans of the brain. Prompt drainage of the abscess and lengthy antibiotics improve the prognosis significantly. Our case highlights a rare origin of subdural empyema from the direct spread of a skin abscess.

## Introduction

Subdural empyema or subdural abscess are also referred to as purulent pachymeningitis, phlegmonic meningitis, and subdural suppuration, and these names can help understand the pathophysiology and the treatment of subdural empyema [1]. Subdural empyema is more common among children than adults and males than females [1]. The infection usually starts in an area near the brain, such as the sinuses, oral cavity, or middle ear, from where it can potentially travel to the brain parenchyma either through emissary veins, hematogenous seeding, or direct invasion following penetrating trauma. About 50-80% of subdural empyemas originate from sinus or ear infections, and around 20% can develop secondary to head trauma or cranial surgery [2]. Significant damage secondary to edema and herniation can occur. Hence, subdural empyema may lead to debilitating consequences and death due to the direct compression and injury of the brain.

Anaerobic bacteria and microaerophilic streptococci (*Streptococcus milleri* and *Streptococcus anginosus*) can cause subdural empyema secondary to sinus infection. Out of *Streptococcus milleri* subgroups, *Streptococcus intermedius* is rare and tends to cause central nervous system (CNS) infections. *Streptococcus intermedius* is not frequently present with a skin abscess compared to *Staphylococcus aureus*, which tends to be a more common intruder with subdural empyema secondary to direct spread from skin infections. Diabetes mellitus (DM) diminishes the immune system through damage to the neutrophil function, depression of the antioxidant system and humoral immunity, endothelial damage, and peripheral neuropathy.

Here, we present a rare case of a diabetic patient found secondary to intractable seizures from subdural empyema caused by *Streptococcus intermedius*. With appropriate investigation and management, the patient recovered successfully.

## Case presentation

**Figure 1:**
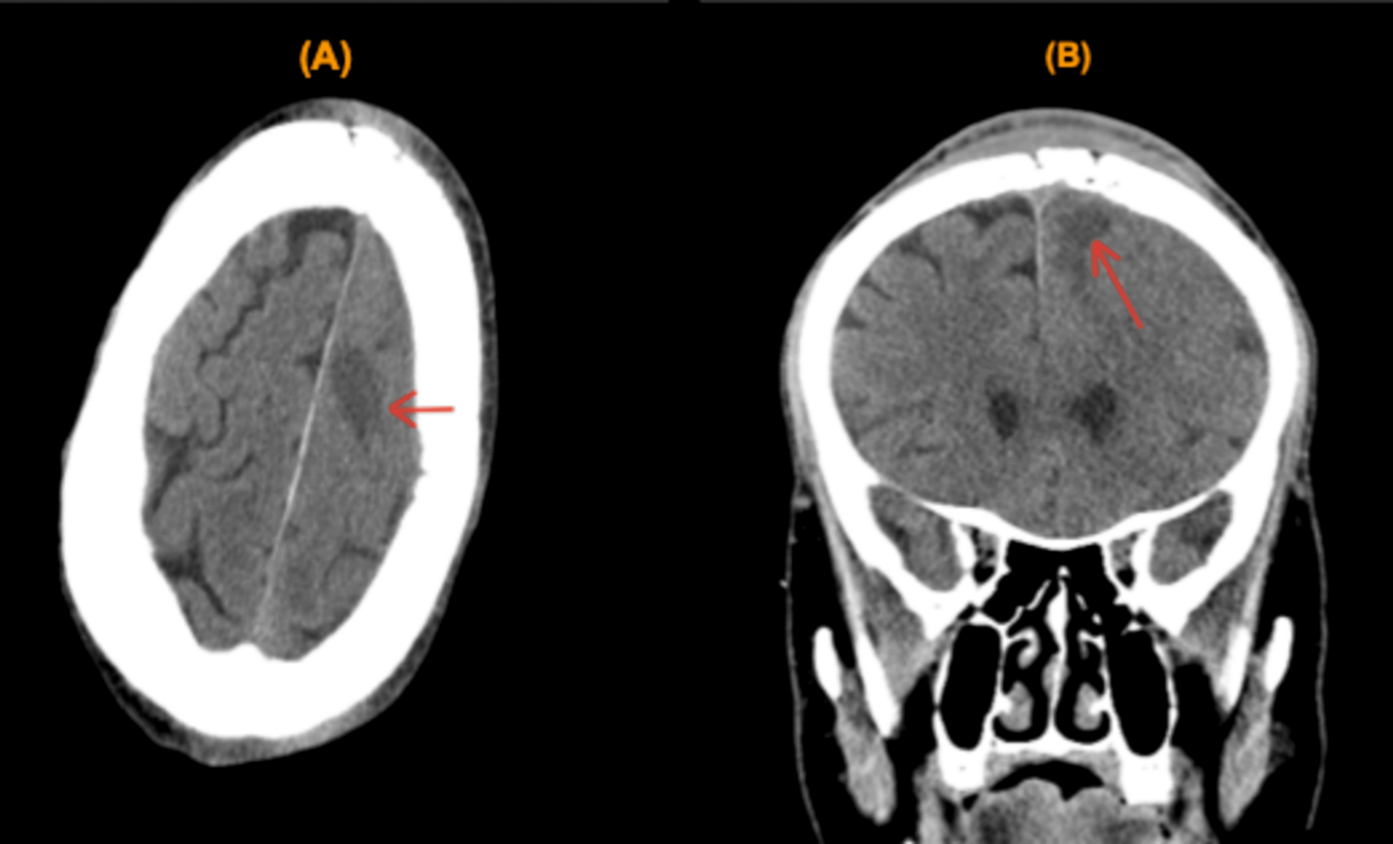
CT scan of the head. (A) Axial plane. (B) Coronal plane. CT scan of the head. (A) Axial plane. (B) Coronal plane. The red arrow points to a lytic lesion near the frontal bone with a midline shift.

**Figure 2:**
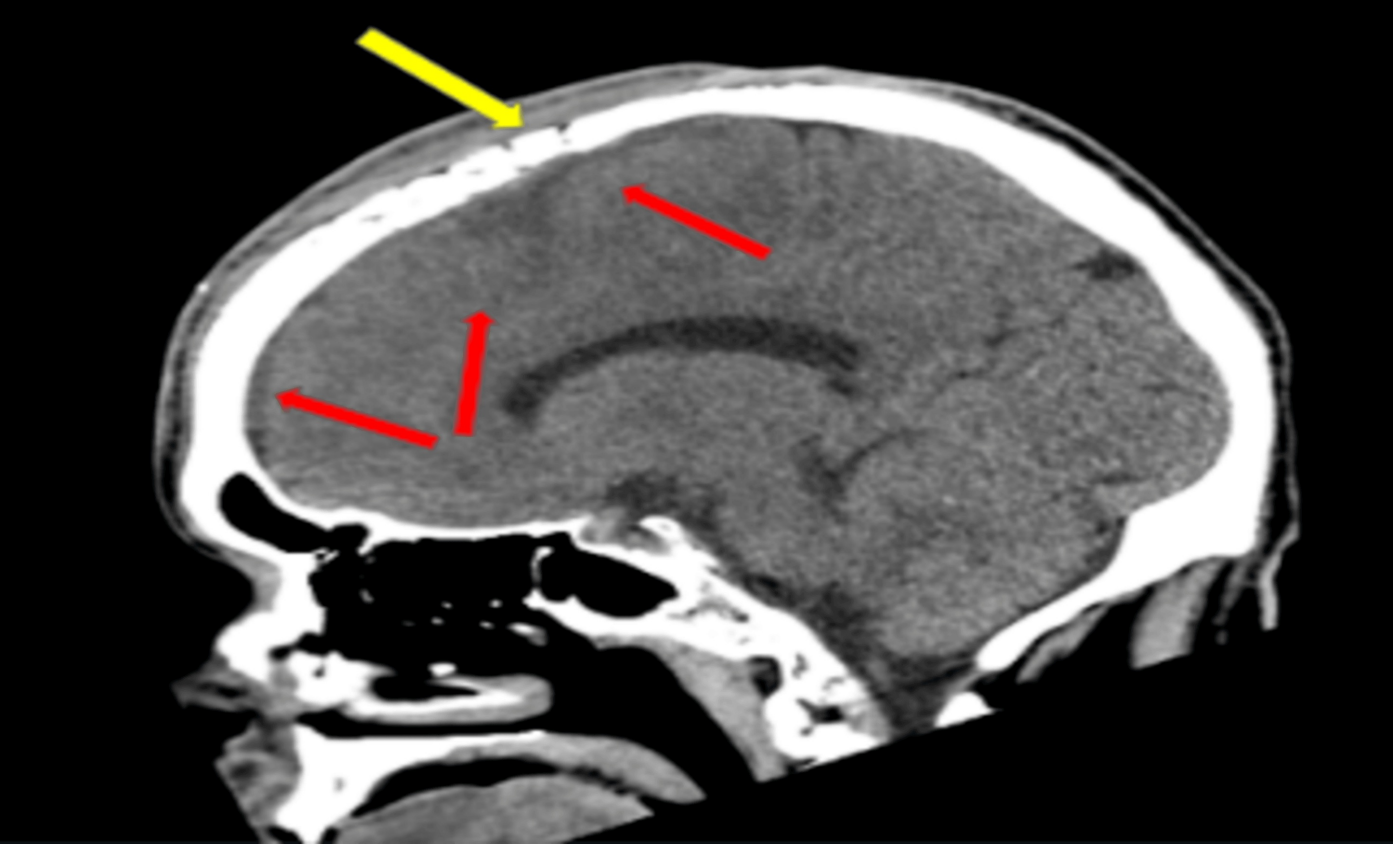
CT scan of the head. Sagittal plane of a CT scan of the head showing the subdural extension of fluid from the lytic lesion (red arrows). Erosion of the skull indicating osteomyelitis (yellow arrows).

A 46-year-old male with a past medical history of uncontrolled DM and polysubstance abuse was brought to the emergency department (ED) by the advanced life support team after his sister found him unresponsive after three episodes of witnessed seizures, which were generalized, involving the whole body according to the rescuers, for the first time in his life according to his family. On route to the ED, the patient developed two more episodes of seizure activity that did not respond to two doses of 2 mg intravenous (IV) lorazepam. On arrival at the ED, the patient was not responding to verbal or painful stimuli with a Glasgow Coma Scale (GCS) score of 8, and right-sided weakness was noted. Immediate intubation was done successfully, and the patient was given a loading dose of 1 g of levetiracetam. With a meticulous review of the patient’s medical chart, the patient presented two months earlier to the ED due to a bump on his head that was red and warm to the touch. However, unfortunately, the patient eloped before being evaluated by a physician. A CT scan of the head showed a lytic lesion near the frontal bone (Figures 1, 2).

Initial blood work revealed blood glucose (300s) with a corrected anion gap of 24 and white blood cells (WBCs) of 25.7; the rest of the labs are presented in Table 1. Critical care service was consulted, and the patient was transferred to the medical intensive care unit (MICU) for further monitoring. With positive ketones, an insulin drip was started for diabetic ketoacidosis.

**Table 1:** Blood work on admission day. Blood work on admission day. As noted, the patient has anion gap metabolic acidosis with low levels of bicarbonate and positive ketones. HbA1C is abnormally elevated for uncontrolled diabetes mellitus. Leukocytosis with high white blood cell count.

Labs	Value	Reference range
Sodium	143 mEq/L	135–145 mEq/L
Potassium	3.3 mEq/L	3.5–5 mEq/L
Chloride	103 mEq/L	98–107 mEq/L
Bicarbonate	18 mEq/L	21–31 mEq/L
Glucose	310 mg/dL	70–110 mg/dL
Ketones	Positive	Negative
Blood urea nitrogen	15 mg/dL	7–23 mg/dL
Creatinine	0.56 mg/dL	0.6–1.3 mg/dL
Estimated glomerular filtration rate	>60 mL/minute/1.73 m^2^	>60 mL/minute/1.73 m^2^
Bilirubin total	0.6 mg/dL	0.3–1.1 mg/dL
Total protein	5.7 g/dL	6.4–8.4 g/dL
Albumin	2.8 g/dL	3.5–5.7 d/dL
Alkaline phosphate	74 U/L	34–104 U/L
Aspartate transaminase	11 U/L	13–39 U/L
Alanine transaminase	8 U/L	7–52 U/L
Hemoglobin A1c	9.8%	4–6%
Magnesium	1.8 mg/dL	1.7–2.5 mg/dL
C-reactive protein	193.7 mg/L	<9.9 mg/L
Erythrocyte sedimentation rate	82 mm/hour	<10 mm/hour
White blood cell count	25.7 × 10^3^ cells/mm3	4.5–11 × 10^3^ cells/mm3
Red blood cell count	3.5 × 10^6^ cells/mm3	4.3–5.8 × 10^6^ cells/mm3
Hemoglobin	10.1 g/dL	13.5–17.5 g/dL
Hematocrit	31.2%	41-53%
Platelets	526 × 10^6^ cells/mm3	140–440 × 10^6^ cells/mm3
Bands	2%	0–10%

**Figure 3:**
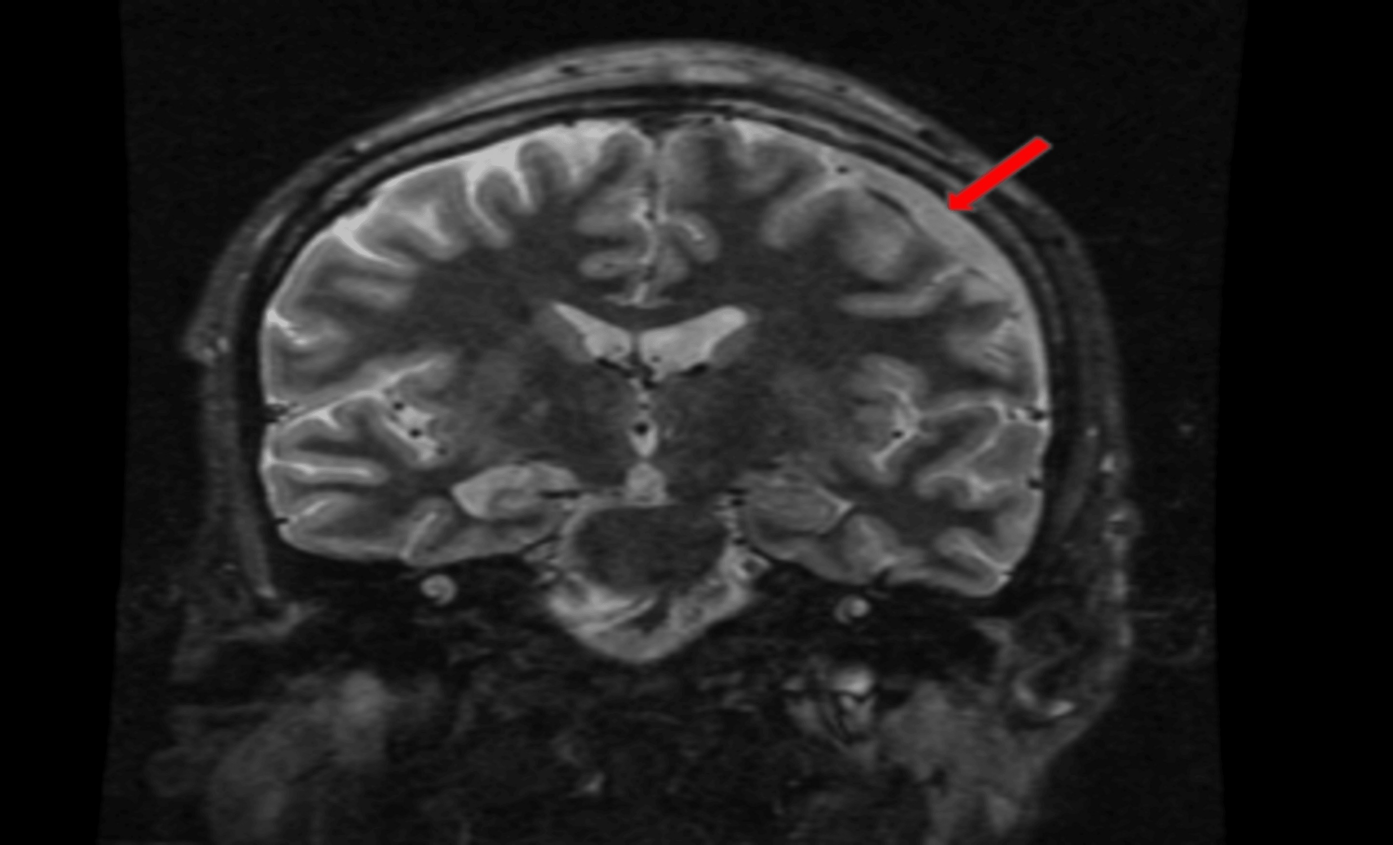
MRI of the brain. MRI of the brain showing an enhancing lesion in the subdural space (red arrows). Differential diagnoses are intracranial bleeding versus subdural empyema.

Before landing in the MICU, a stat MRI of the brain was notable for enhancing lesions with either intracranial bleeding or subdural empyema (Figure 3), and neurology and neurosurgical consults were placed. In the MICU, cefepime and vancomycin were started. The infectious disease specialist recommended starting amphotericin B for possible mucormycosis due to uncontrolled DM and metronidazole for anaerobic coverage. The electroencephalogram (EEG) showed diffuse background slowing and attenuation, suggesting severe encephalopathy with no evidence of active seizure or epileptogenic potential. Neurosurgery evaluated the patient in the MICU and decided to take the patient to the operating room (OR) immediately as he interpreted the MRI as subdural empyema rather than a hemorrhage.

In the OR, a bicoronal craniotomy was performed to drain the pus. There was significant difficulty releasing the pericranium from the skull due to significant osteomyelitis in the midline skull where the bone was eroded, and there was phlegmon extending from the skull into the galea. The neurosurgeon placed a burr hole in the inferior temporal region, placed burr holes on either side of the superior sagittal sinus all the way posteriorly to be able to identify the end of the bony defect from the osteomyelitis, and then turned a craniotomy flap over the left frontotemporoparietal region. Then, he attempted a second craniotomy flap extending over the superior sagittal sinus toward the right side. The bone was sent to microbiology and pathology. As the bone was being removed, the bone was splitting in half, and there was pus coming from the inner table. As the dura opened up, large amounts of pus within the subdural empyema were evacuated and sent to microbiology and pathology. After careful pus evacuation, irrigation was done with copious amounts of antibiotics in the subdural space until only clear fluid returned. The patient was vitally and clinically stable after the surgery and was transferred to the surgical intensive care unit (SICU).

In the SICU, the wound cultures grew *Streptococcus intermedius*, and the insulin drip was stopped with the resolution of diabetic ketoacidosis. On the fifth day of admission, the patient was successfully extubated and gradually started to respond to verbal stimulation. The fever resolved, WBCs smoothly trended down, and amphotericin B was discontinued with negative fungal growth in wound and blood cultures and normal fungitell levels. Sensitivity was positive for cefazolin, and the patient was kept only on IV cefazolin. After seven days in the SICU, no seizure activity was recorded on the EEG, and the patient was moved to the medical ward. Seven days later, the patient was successfully discharged to the acute rehabilitation center with a continuation of IV cefazolin for six to eight weeks. Two months later, the patient appeared in good health in the infectious disease clinic and reported no recurrent seizures or neurological deficit.

## Discussion

CNS infections are potentially lethal, either intracranially or extracranially. Intracranial bacterial infections are uncommon, and yet life-threatening, and include brain abscess and subdural or extradural empyema, and are classified according to the anatomical location or the etiologic agent. Intracranial infections can be classified as focal (e.g., brain abscess, epidural abscess, and subdural empyema) or diffuse (e.g., pyogenic meningitis or ventriculitis) [3]. Subdural empyema occurs less frequently than brain abscess and recurs less often in the spinal column [4]. Subdural empyema etiology is multifactorial. Due to the valveless diploic veins of Breschet, the direct spread of local infection or blood spread of distant infection in either direction is noted. Although focal, subdural empyemas can spread over the convexity of the brain between both cerebral hemispheres and, in some cases, to the opposite hemisphere or even the posterior fossa due to a lack of anatomical barrier in the subdural space [5]. Paranasal sinusitis is the most common predisposing condition that leads to subdural empyema, which could start extracranially through osteomyelitis of the frontal bone, i.e., Pott’s puffy tumor [6]. Bacteremia may or may not be present with abscess in patients with infection secondary to hematogenous seeding, making the diagnosis complex. Bacteremia did not develop in our patient as the brain abscess was caused by direct skin and skull invasion.

Streptococci and staphylococci account for most bacteria cultured from pyogenic brain infections. Meanwhile, streptococci are associated more often with brain infections originating from distant foci and spreading through the blood [7]. Of all pathogenic bacteria causing CNS infections, streptococci have been isolated in 30-60% of cases [8]. *Streptococcus anginosus* includes three bacteria, namely, *Streptococcus anginosus*, *Streptococcus constellatus*, and *Streptococcus intermedius* [9]. While *Streptococcus anginosus* is more often associated with intra-abdominal and gastrointestinal tract infections, *Streptococcus intermedius* is more inclined to cause purulent head, neck, and CNS infections [10]. *Streptococcus intermedius* is a commensal oral flora in humans. Therefore, dental procedures can lead to bacteremia and hematogenous seeding in distant organs without an active oral infection [11]. *Staphylococcus aureus* is also a common culprit in oral cavity infections [12]. *Streptococcus intermedius* has different virulence factors that induce tissue destruction and facilitate abscess formation; for instance, a polysaccharide capsule, which helps the bacteria avoid phagocytosis, can capsulize into a collection of pus and form an abscess. In addition, it can produce hydrolytic enzymes such as hyaluronidase, which, in collaboration with biofilm formation and superantigen activation, can induce abscess formation [13].

Multiple risk factors can affect the presentation of subdural empyema, such as infection origin, virulence of bacteria, and immune status. Common symptoms include fever, obstructive symptoms (headache, nausea, vomiting), focal neurological deficits, seizures, and mental status changes. Depending on meningeal involvement, a stiff neck and photophobia may be present. Although subdural empyema can be asymptomatic, some patients may promptly develop lethargy and deep coma. With limited space to swell, increased intracranial tension can yield Cushing’s triad of hypertension, bradycardia, and bradypnea. Other non-neurological symptoms can also be present and can aid in diagnosing subdural empyema, such as nasal discharge, sore throat, sinus pain, and pressure. The neurological presentation in our case (seizure) pointed the diagnosis toward a CNS culprit.

DM increases the odds of acquiring infections. Moreover, DM can halt the immune system from fighting, resulting in more extended hospital stays and complications. Skin infections, such as abscesses or cellulitis, can rapidly spread to the bone, causing osteomyelitis due to the poor blood supply to peripheral tissues, which affects the delivery and full function of leukocytes and lymphocytes to sites of bacterial infections. DM is notorious for causing frequent foot infections, malignant external otitis, and rhinocerebral mucormycosis, among others. Hence, anti-pneumococcal and influenza vaccines are recommended for diabetic patients to reduce hospitalizations, deaths, and medical expenses. DM is a well-known risk factor for developing persistent infections due to suppressed immunity. A seven-year retrospective analysis calculated the DM risk factor as 18% for developing* Streptococcus anginosus* group infection after having a solid tumor as the highest risk factor at 32% [14]. Our patient has uncontrolled DM, and with both micro- and macroangiopathies and neuropathy, the small skin pimple extended to the skull, causing osteomyelitis and subdural empyema.

Most odontogenic infections originating from the oral cavity involve cavernous sinus thrombosis and can also cause isolated brain abscesses and subdural empyema [15-17]. Cardiac causes of brain abscesses should be sought in patients with unclear risk factors. The presence of right to left shunt, as in patent foramen ovale and other congenital anomalies, has been reported as the cause of brain abscesses [18]. Not only brain involvement but *Streptococcus intermedius* involving the spinal cord and forming an abscess has been reported [19], which was attributed to the presence of right to left shunt. CT angiography identified the right superior vena cava draining into the left atrium (which is an uncommon systemic venous anomaly) that results in a right to left shunt, causing the intramedullary spinal cord abscess. *Streptococcus intermedius* is also found in normal urogenital flora. Brain abscesses should be considered in females with intrauterine devices and no risk factors. A metastatic brain abscess has been reported in pelvic inflammatory disease secondary to *Streptococcus intermedius*-mediated infection of an intrauterine device [20].

In 2020, Elio et al. [21] reviewed 101 cases of different *Streptococcus intermedius* infections, including brain and liver abscesses, and concluded that the recovery of all cases, based on statistical calculations, was around 92%, while the death rate was roughly 8%. In their analysis, the most common primary symptoms were intermittent fever and consistent headaches. The main risk factors for developing *Streptococcus intermedius* infections were dental manipulations (18%), sinus infection (12%), alcohol consumption (8%), and heart disease comorbidity (8%). Our case highlights the rarity of a skin infection that was left untreated and migrated through the skull, causing osteomyelitis and subsequent brain abscess. Out of the 101 cases, *Streptococcus intermedius* was isolated from the brain in 17 cases and from the cerebrospinal fluid in seven cases. Imaging detection by CT and MRI scans of *Streptococcus intermedius* infections was very reliable, as almost all infections were associated with abscess formation. Elio et al. [21] also analyzed the treatment course of most cases in detail. They highlighted the importance of either abscess drainage or surgery as the gold standard interventions to control the source of the infection. Various antibiotics were given to the patients, and combination antibiotic therapy was the most commonly used approach. Ceftriaxone and metronidazole alone or combined with vancomycin were most frequently used initially, and then the antibiotic choices were changed to include metronidazole, ceftriaxone, or meropenem. *Streptococcus intermedius* can also cause brain abscesses, usually as a single lytic lesion. However, multifocal brain abscesses have been reported and are precipitated by *Streptococcus intermedius*. Brain abscess infection can present suddenly or in an indolent fashion. The variable onset of presentation depends on many factors, such as size, location, and immune status. Neurologic symptoms usually manifest early, with the main complaint being headache. However, altered mental status, motor deficit, and seizure can also present initially. Hydrocephalus and intracranial hypertension can also occur with brain abscesses causing cerebrospinal fluid obstruction. The invasive feature of *Streptococcus intermedius* can help isolate the bacteria in blood culture; however, it is not a general rule of thumb as bacteremia may not occur even in hematogenous spread with *Streptococcus intermedius* brain abscess.

## Conclusions

Subdural empyema is a gruesome infection that needs hypervigilant diagnosis, evaluation, and treatment. Most cases involve men under 60 years of age and are rarely seen in children. It results from a near or distant bacterial infection that spreads to the subdural space. While different groups of bacteria can cause it, gram-positive staphylococci and streptococci are significant culprits. If it becomes invasive, *Streptococcus intermedius* infections have significantly higher mortality rates than other streptococci. Dental manipulation or sinusitis are the two most important underlying risk factors for *Streptococcus intermedius* infections, with skin abscesses being rare. Despite this rarity, our patient developed a small skin abscess that caused osteomyelitis of the frontal bone and further spread deeper to cause subdural empyema. With emergent drainage, a lengthy course of antibiotics, and comprehensive care, the patient fully recovered. Our case highlights the importance of a detailed history investigation, considering rare causes of seizure and subdural empyema, and the proper management of such cases.
